# La responsabilité médicale en obstétrique au Maroc: analyse des dossiers des contentieux judiciaires entre 2015-2018

**DOI:** 10.11604/pamj.2022.43.150.26376

**Published:** 2022-11-22

**Authors:** Ikram Boubess, Rachid Bezad, Mohammad Hassan Alami

**Affiliations:** 1 Hôpital Maternité Santé de Reproduction les Orangers, Centre Hospitalier Universitaire Ibn Sina, Rabat, Maroc,; 2 Laboratoire de Biostatistique et de Recherche Clinique et Biologique Université Mohammed V, Rabat, Maroc

**Keywords:** Responsabilité obstétricale, qualité de l’expertise, indemnisation, aléa thérapeutique, Obstetric liability, expertise quality, compensation, therapeutic hazard

## Abstract

Les réflexions en matière de la responsabilité médicale au Maroc sont en nette développement. Les professionnels de la santé et spécialement les obstétriciens réclament une situation désastreuse entre des ressources très limitées et l´absence de cadre juridique clair qui les protègent avec l´inflation des litiges ces dernières années. Nous avons collecté les dossiers des contentieux obstétricaux ayant un jugement au niveau du service des contentieux du ministère de la santé au Maroc sur une période de trois ans (2015-2018), dans le but d´évaluer la situation actuelle de la responsabilité médicale en Obstétrique et de répertorier les défaillances du traitement des contentieux obstétricaux et afin d´établir des recommandations adaptées au contexte marocain. Parmi les cent et onze contentieux médicaux ayant un jugement durant cette période, 56% contentieux (n=62) ont concerné l´activité obstétricale, les complications de la période du travail sont trouvées dans 84% des cas (n=54), le plexus brachial est le sujet de plainte le plus enregistré 61,3% (n=39). L´analyse de l´expertise médicale dans ces dossiers objective la non-conformité à la qualité dans 88,7% des cas (n=55), la condamnation des professionnels accoucheurs est trouvée dans 93,5% (n= 58), le manque de la documentation dans le dossier médical est trouvé dans 35% des cas (n=22). Les litiges judiciaires sont plus enregistrés en obstétrique que dans les autres domaines du secteur de santé publique au Maroc. Le manque de la documentation et la complexité du circuit du traitement des contentieux médicaux ainsi que la tendance à l´indemnisation des patientes en absence d´une structure chargée de la prise en charge des victimes d´ aléa thérapeutique sont les causes les plus fréquentes du gain des demandeurs.

## Abstract (en)

Reflections on professional responsibility in Morocco are in clear development. Health professionals and especially obstetricians are reporting a dire situation due to limited resources and the lack of a clear legal framework that protects them, given that litigations are on the rise in recent years. We collected medical records of obstetric litigations adjudicated at the litigation department of the Ministry of Health in Morocco over a period of three years (2015-2018), with the aim of assessing the current situation of professional responsibility in obstetrics and indentifying failures in obstetric litigation management as well as developing recommendations tailored to the Moroccan context. Among the 111 medical litigations adjudicated over the study period, 56% (n=62) concerned obstetric activity and 84% (n=54) complications during labor; brachial plexus was the most registered complaint (61.3%; n=39). The analysis of data on medical expertise from these medical records revealed a non-compliance with quality in 88.7% of cases (n=55), the condemnation of professional birth attendants in 93.5% (n=58), a lack of documentation in the medical records in 35 % of cases (n=22). Litigations are more recorded in obstetrics than in other areas of the public health sector in Morocco. The lack of documentation and the complexity of management of the litigation process as well as the tendency to compensate patients in the absence of a structure of medical care for victims of therapeutic hazards are the most frequent causes of claimants´ gain.

## Introduction

La gynécologie obstétrique est le domaine de médecine le plus exposé aux critiques sociaux et au recours à la justice au monde [1]. Les obstétriciens sont les professionnels de la santé les plus susceptibles d´être poursuivis devant la justice [2-4], le collège américain des gynécologues obstétriciens dans une étude nationale a déclaré que plus de 60% des réclamations sont en relation avec la période du travail et de l´accouchement [5]. En France, les sinistres corporels en obstétrique constituent 45% des réclamations de l´assurance hospitalière des naissances [6]. Au Maroc, comme en occident, les problèmes médico-légaux sont de plus en plus soulevés, avec l´évolution socioculturelle des citoyens marocains et la médiatisation importante des accidents médicaux surtout en rapport avec l´obstétrique. Parallèlement à ce recours, se sont développées des réflexions dans les congrès, les colloques et les publications nationales sur la problématique de la responsabilité médicale dans notre pays et l´absence du cadre juridique adapté à notre contexte de ressources limitées. Pourtant, il est difficile d´évaluer avec précision la situation au Maroc en absence des données objectives sur le sujet. Dans ce cadre, on a voulu mettre le point sur la situation de la responsabilité médicale en obstétrique au Maroc par l’analyse des dossiers des contentieux judiciaires portés sur les médecins marocains du secteur publique et spécialement les obstétriciens pendant une période de trois ans (2015-2018), afin de cerner objectivement cette problématique.

## Méthodes

**Plan d´étude:** il s´agit d´une étude rétrospective descriptive et analytique des contentieux obstétricaux du secteur public qui ont eu un jugement dans les tribunaux 1^er^ janvier 2015 au 31 décembre 2018. On a collecté ces dossiers des archives du service des contentieux et des affaires juridiques du ministère de la santé au Maroc qui s´en charge du suivi des contentieux portés contre les professionnels de la santé publique.

**Source de données:** on a consulté ces dossiers constitués d´une copie du dossier médical sujet de litige et /ou le rapport médical, du rapport de l´expertise et du rapport du jugement sur le litige. On a d´abord recensé tous les dossiers des litiges médicaux enregistrés durant cette période, puis on a sélectionné les contentieux en obstétrique pour une analyse approfondie: l´intégrité du dossier du contentieux, profil des patientes (âge, parité, suivi de grossesse), motif de plainte, phase du travail à l´admission, prise en charge de la complication par les professionnels, l´analyse de l´expertise (spécialité de l´expert, conformité aux données scientifiques), le résultat du jugement (montant d´indemnisation, compatibilité avec l´expertise, délai pour jugement).

**L´analyse statistique:** on a enregistré ces données dans un tableau Excel et les résultats sont exprimés en nombre, en moyenne et en pourcentage. On n´a pas utilisé de test spécifique pour l´interprétation des résultats puisqu´il s´agit d´une étude plutôt descriptive qui va permettre l´analyse objective et spécifique des litiges obstétricaux au Maroc tout en exposant la situation d´autres pays en matière de la responsabilité médicale.

**Considération éthique:** on souligne qu´on a été autorisé à exploiter ces dossiers après avoir adressé une demande manuscrite à la direction des contentieux et des affaires juridiques du ministère de la santé marocaine à Rabat.

## Résultats

### Données générales

On a recensé 111 plaintes judiciaires durant cette période dont 80,7% (99 dossiers) porté directement contre les professionnels de la santé. Pour ces contentieux (n=99) l´obstétrique constitue le premier domaine visé par les demandeurs avec un taux de 62,6 % (n=62), suivi par les autres spécialités chirurgicales (confondues) avec un taux 31,3% (n=31), tandis que les spécialités médicales n´étaient concernées que dans 7% des cas (n=6).

### Les contentieux obstétricaux

L´analyse des 62 contentieux collectés durant cette période objective pour le profil des patientes: un âge des patientes qui varie entre 18 ans et 42 ans, avec un âge moyen de 32,3 ans. La parité des patientes varie entre 1 et 7 avec une parité moyenne de 2,62. Dans 65% (n=40) des cas, les patientes n´ont pas suivi leur grossesse ou elles ont eu un suivi incomplet, on a recensé 16% (n=10) des grossesses jugées à risque (obésité, diabète). On constate que 84% (n=52) des plaintes ont concerné les complications de la période du travail. Parmi les 36 dossiers dont on possède l´information sur la phase du travail, 14 parturientes ont été admises à une phase avancée du travail (9 patientes admises à dilatation complète ou en expulsion). Le plexus brachial constitue 61,3% (n=38) des motifs de plainte comme l´objective le Tableau 1.

**Tableau 1:** répartition des dossiers en fonction des plaintes

Motif de la plainte	Nombre des dossiers	pourcentage
Plexus brachial	39	61,3%
Retard psycho moteur après accouchement prématuré	1	1.6%
Retard psychomoteur et développement d´une microcranie après expulsion du nouveau-né par terre lors d´accouchement	1	1.6%
Textilome	1	11.3%
Plaies vésicales et rectales	2	3.2%
Asphyxie néonatale	3	4.8%
Décès maternel	5	8%
Hystérectomie d´hémostase	2	3.2%
Prolapsus 2^e^ degrés après accouchement	1	1.6%
Fracture du pied après une césarienne	1	1.6%

Le manque de la documentation dans le dossier du contentieux (hôpital concerné contacté hors délai ou dossier médical perdu) est rapporté dans 35% des cas (n=22), le juge a fait recours à l´expertise dans 93,5% des plaintes (n=58) tandis que pour les autres quatre dossiers, les juges ont considéré que la faute du service est évidente, le Tableau 2 montre le domaine des experts qui ont été consultés dans ces contentieux obstétricaux. On remarque que le recours à des experts de plusieurs spécialités autre que l´obstétrique ainsi qu´aux généralistes est une pratique courante dans le traitement des contentieux obstétricaux. Le rapport de l´expertise était en faveur des demandeurs dans 88,7% des cas (n= 55). Concernant le jugement, la demande est jugée en faveur des demandeurs dans 93,5% des cas (n=58) et rejetée dans seulement 6,5% (n=4). Le délai entre la plainte et le jugement a varié entre 96 mois et 6 mois avec un délai moyen de 17 mois.

**Tableau 2:** répartition des dossiers en fonction du domaine de l’expert

Domaine de l´expert	Nombre	Pourcentage
Gynécologie obstétrique	14	22,5%
Traumatologie	6	9,6%
Rhumatologie	3	4,8%
Chirurgie générale	6	9,6%
Médecine légale	1	1,6%
Médecine générale	14	22,5%
Neurochirurgie	1	1,6%
Pédiatrie	1	1,6%
Urologie	1	1,6%
Non précise	9	14 ,5%
Médecine de travail	2	3,2%

## Discussion

L´accouchement est une situation physiologique qui peut, malheureusement, se compliquer dans 15% des cas [7]. Ces complications déclenchent parfois des litiges contre les prestataires des soins et même le recours à la justice. En réalité, les poursuites judiciaires résultent de seulement de moins de 0,1% des naissances selon une étude britannique [8], en outre il a été démontré, dans les cas par exemple des infirmités motrices présumées d´origine obstétricale, que la mauvaise prise en charge de l´accouchement est à l´origine de moins de 10% des séquelles neurologique [9]. Néanmoins, on observe une médiatisation large des accidents obstétricaux au monde, comme dans notre pays, ce qui a abouti à une inflation des indemnisations dans le domaine de la responsabilité obstétricale dans les pays occidentaux. Cette nouvelle situation a créé une vraie crise dans certains Etats surtout américains où certains obstétriciens ont commencé à fuir cette discipline et une question qui s´est déclenchée: qui va faire naitre nos petits enfants? [10]. Au Maroc, cette problématique est de plus en plus soulevée malgré l´absence des données objectives sur le sujet. Notre étude nous a permis de constater que le taux le plus élevé des litiges médico-légaux est enregistré dans le domaine obstétrical, le traitement des contentieux qui en résultent connait plusieurs lacunes dont la plus importante est la non-conformité de l´expertise médicale aux données scientifiques et la tendance à l´indemnisation des victimes en absence de faute médicale.

Dans plusieurs pays comme la France, on assiste à une dépénalisation du droit médical [11], mais avec un élargissement du champ de la responsabilité médicale et de la responsabilité administrative de certains services hospitaliers en cas de préjudice sans faute lié à l´aléa thérapeutique. Dans notre étude, on s´est intéressé à la responsabilité indemnitaire dont le circuit est bien établi et qui pèse sur le budget alloué au secteur de la santé au Maroc qui ne constitue que 5,9% du produit interne brut [12]. Dans notre contexte, l´évolution jurisprudentielle des dossiers des contentieux médicaux est majoritairement défavorable vis-à-vis les prestataires du soin, ceci est lié au manque de document dans le dossier médical qui est une cause commune de perte du contentieux devant les tribunaux [3]: 35% des cas dans notre étude. Elle est aussi liée à la qualité de l´expertise obstétricale [13], une bonne expertise médicale ne peut être garantie que si on exige une expertise basée sur les niveaux de preuve-evidence *based medicine* [14] ou en suivant les recommandations des sociétés savantes si elles existent [13]. Alors que l´expertise médicale au Maroc est faite majoritairement par des non obstétriciens (77,41% des cas selon notre étude), il semble que ce constat est loin de processus de qualité. D´autre part, d´autres facteurs interviennent dans notre contexte pour balancer le jugement en faveur du demandeur en dehors de l´acte de soin en lui-même. La complexité du circuit de correspondance entre les différents services qui traitent ces contentieux et le risque de réception des réponses au tribunal hors délai, en plus de la charge des dossiers à traiter par l´agence judiciaire du royaume chargée de défendre les fonctionnaires de l´Etat. Aussi on peut citer l´inadéquation de l´offre du soin dans le service public au Maroc avec la demande croissante d´une population démunie, s´ajoutant à une pénurie cruelle en obstétriciens est une autre source évidente de la mauvaise gestion des malades et donc de litiges [15].

Ces facteurs affectent évidemment la jurisprudence marocaine, on constate que les juges ont tendance à indemniser les victimes en jugeant en leur faveur sans prendre en considération la part de responsabilité des patientes (grossesse non suivie, arrivée à dilatation avancée ou accouchement imminent), même si le praticien a agi dans les règles de l´art selon l´expert (6 dossiers dans notre série): les juges sont souvent influencés par la souffrance de ces victimes, en absence totale des structures qui vont s´en charger des patients qui ont subi un tel sinistre. Ce point a été un objectif essentiel de la loi « Kouchner » adoptée en 2002 en France qui a permis d´éviter aux juges d´imputer à la responsabilité du médecin en se préoccupant à indemniser la victime [16]. Malgré que notre travail nous a permis concrètement de soulever ces reproches en matière de la responsabilité obstétricale, mais cette étude reste limitée par le manque d´information sur la prise en charge des complications lors de l´accouchement dans plusieurs dossiers de ces contentieux. Aussi on n´a pas pu cerner la situation totale au Maroc vu la taille de l´échantillon et l´absence de données sur le secteur libéral. Mais vu le retentissement économique élevé de ce problème, nous invitons le ministère de la santé à agir pour minimiser ce coût par l´installation d´une politique de prévention en mettant en place un processus de qualité et par la normalisation des structures et du circuit du soin, le recrutement suffisant des praticiens et la formation continue des professionnels de la santé car il a été démontré que la formation des professionnels en urgences obstétricales améliore la qualité de prise en charge et diminue le taux des litiges médicolégales [17-20]. Les obstétriciens doivent aussi se préparer au litige en cas d´événement non souhaitable [21] par le maintien d´une communication claire avec les patientes et leurs familles: une bonne relation médecin patient augmente les chances d´éviter les litiges [1]. En cas de litige, nous invitons à avoir une commission d´étude de ces dossiers, chargée des indemnisations des victimes en cas d´aléa thérapeutique ou d´erreur médical sans recours à un avocat comme dans les pays occidentaux. Cette procédure évitera au professionnel de santé le sentiment d´injustice dans la procédure judiciaire en cas d´aléa thérapeutique.

## Conclusion

En matière de responsabilité médicale au Maroc, les litiges en obstétrique sont les plus fréquemment enregistrés et les poursuites juridiques qui en résultent sont majoritairement en faveur des demandeurs. Plusieurs facteurs sont incriminés dans cette évolution jurisprudentielle défavorable pour les obstétriciens dont l´expertise non basée sur les données scientifiques, la perte des documents du dossier médical ainsi que la tendance à l´indemnisation des victimes par les juges en cas d´aléa thérapeutique. La protection de poursuite judiciaire est devenue une revendication principale des professionnels de la santé, spécialement les obstétriciens, et l´adoption des mesures préventives dans notre contexte de pauvreté est la première étape pour faire face à cette problématique.

### Etat des connaissances sur le sujet

- La crise de la responsabilité médicale des obstétriciens est en explosion dans le monde;
- Le développement de la médecine défensive en obstétrique retentit sur la santé maternelle et l´économie de la santé;
- La jurisprudence dans la responsabilité médicale est très évoluée dans les pays occidentaux.

### Contribution de notre étude à la connaissance

- Au Maroc, les poursuites judiciaires sont plus enregistrées en obstétrique que dans les autres domaines;
- Le manque de la documentation et la mauvaise expertise médicale sont les facteurs de gain des demandeurs;
- Les juges tendent à indemniser les victimes de l´aléa thérapeutique.
